# Anti-Inflammatory, Analgesic, Functional Improvement, and Chondroprotective Effects of *Erigeron breviscapus* (Vant.) Hand.-Mazz. Extract in Osteoarthritis: An In Vivo and In Vitro Study

**DOI:** 10.3390/nu16071035

**Published:** 2024-04-02

**Authors:** Hee-Geun Jo, Chae Yun Baek, JunI Lee, Yeseul Hwang, Eunhye Baek, Ji Hye Hwang, Donghun Lee

**Affiliations:** 1 Department of Herbal Pharmacology, College of Korean Medicine, Gachon University, 1342 Seongnamdae-ro, Sujeong-gu, Seongnam-si 13120, Republic of Korea; jho3366@hanmail.net (H.-G.J.);; 2 Naturalis Inc., 6, Daewangpangyo-ro, Bundang-gu, Seongnam-si 13549, Republic of Korea; 3 RexSoft Inc., 1 Gwanak-ro, Gwanak-gu, Seoul 08826, Republic of Korea; 4 Department of Acupuncture and Moxibustion Medicine, College of Korean Medicine, Gachon University, 1342 Seongnamdae-ro, Sujeong-gu, Seongnam-si 13120, Republic of Korea

**Keywords:** east Asian herbal medicine, osteoarthritis, *Erigeron breviscapus*, anti-inflammatory, analgesic, chondroprotective

## Abstract

Osteoarthritis (OA) is a degenerative bone disease characterized by inflammation as a primary pathology and currently lacks therapeutic interventions to impede its progression. *Erigeron breviscapus* (Vant.) Hand.-Mazz. (EB) is an east Asian herbal medicine with a long history of use and a wide range of confirmed efficacy against cardiovascular and central nervous system diseases. The purpose of this study is to evaluate whether EB is worthy of further investigation as a treatment for OA based on anti-inflammatory activity. This study aims to assess the potential of EB as a treatment for OA, focusing on its anti-inflammatory properties. Analgesic effects, functional improvements, and inhibition of cartilage destruction induced by EB were evaluated in acetic acid-induced peripheral pain mice and monosodium iodoacetate-induced OA rat models. Additionally, the anti-inflammatory effect of EB was assessed in serum and cartilage tissue in vivo, as well as in lipopolysaccharide-induced RAW 264.7 cells. EB demonstrated a significant alleviation of pain, functional impairment, and cartilage degradation in OA along with a notable inhibition of pro-inflammatory cytokines, including interleukin-1β, interleukin-6, matrix metalloproteinases 13, and nitric oxide synthase 2, both in vitro and in vivo, in a dose-dependent manner compared to the active control. Accordingly, EB merits further exploration as a potential disease-modifying drug for OA, capable of mitigating the multifaceted pathology of osteoarthritis through its anti-inflammatory properties. Nonetheless, additional validation through a broader experimental design is essential to substantiate the findings of this study.

## 1. Introduction

Osteoarthritis (OA) is the most common form of arthritis in adults and is characterized by chronic pain, loss of physical function, and progressive joint destruction [1]. A recent global burden study reported that by 2020, 595 million people worldwide were expected to be living with the disease [2]. The number of patients and the demand for treatment are expected to increase rapidly in the future, as there is currently no effective treatment for OA and the aging of the population is a global trend [1,2,3]. The development of safe and effective treatment options for OA is a public health challenge, as it is the leading age-related bone disease and contributes significantly to the societal burden [4]. However, the greatest unmet medical need is the current lack of a cure for OA and measures to slow the progressive pathology [5]. This is likely due to the complexity of the disease and its still incompletely understood pathogenesis.

OA is no longer viewed as a degenerative disease caused by prolonged mechanical stimulation. Recent studies have emphasized that OA is a multifaceted pathophysiology with complex factors, the most prominent of which is inflammaging [3,6]. This refers to a state of continuous and progressively increasing inflammation throughout the body as we age. This systemic low-intensity inflammatory state leads to joint pain and deterioration, as well as irreversible destruction of articular cartilage and subchondral bone [7]. From this perspective, current conservative treatments for OA do not offer options that effectively halt or reverse the inflammatory pathology of OA, especially in the absence of specific treatment. Therefore, it is not surprising that a new class of drugs that may delay the disease’s progression by modulating the multiple inflammation-related pathologies of OA is being actively investigated. However, none of the disease-modifying osteoarthritis drug (DMOAD) candidates in this new class of OA treatments have successfully completed phase III clinical trials or received regulatory approval in any country [8]. For that reason, the search for safe and effective DMOAD candidates that can modulate the disease by inhibiting the inflammatory multipathology of OA is an urgent research topic to address.

In the context of this research topic, the anti-inflammatory activity of natural products is particularly worthy of attention due to their low toxicity, ease of management of long-standing diseases, and multifaceted targeted modulation of inflammation by multiple active components [9,10,11]. In particular, east Asian herbal medicine (EAHM) has accumulated extensive human dosage information with historical context for hundreds of millions of people throughout East Asia and has recently been studied from a modern perspective [12,13,14,15,16,17,18]. Consequently, this group of materials has potential comparative advantages over other DMOAD natural product candidates in terms of safety and efficacy. Although hundreds of EAHMs have been studied as candidates for the treatment of OA, *Erigeron breviscapus* (Vant.) Hand.-Mazz. (EB) is promising in that it has already been studied for efficacy and used as a ethnopharmacological remedy for a wide range of diseases, including cardiovascular, gastrointestinal, respiratory, and metabolic diseases [19,20]. Of particular note, EBs have been shown to possess potent anti-inflammatory properties, and these properties can effectively support disease-suppressing effects in multiple systems of the body, making them ripe for exploration as DMOAD candidates [20]. However, hitherto, the evidence for EBs has been primarily centered on central nervous system and cardiovascular diseases, and there is very little pharmacologic information to determine whether the activity of this EAHM material may be effective in OA, and further exploratory studies are warranted.

In this study, we hypothesized that *Erigeron breviscapus* (Vant.) Hand.-Mazz. extract (EBE) would effectively alleviate symptoms and inhibit cartilage destruction in osteoarthritis (OA) based on its natural product-specific multi-target anti-inflammatory pharmacology. To test this hypothesis comprehensively, we assessed the effects of EBE on various biochemical markers, inflammatory statuses, and morphological features of rat knee OA induced by monosodium iodoacetate (MIA). Additionally, to anticipate and validate potential modes of action, we examined the analgesic effects in an acetic acid-induced mouse writhing model and elucidated the pathophysiology of OA through in vitro assays involving various inflammatory cytokines and catabolic indicators. The findings of this study support the candidacy of EBE as a potential disease-modifying osteoarthritis drug (DMOAD), as detailed below.

## 2. Materials and Methods

### 2.1. Preparation of Erigeron breviscapus Extract (EBE)

The root of *Erigeron breviscapus* (Vant.) Hand.-Mazz. was purchased from Yaksudang Pharmaceutical Co., Ltd., (Seoul, Republic of Korea). Professor Donghun Lee from the Department of Herbal Pharmacology, College of Korean Medicine, Gachon University, deposited a voucher specimen (D211124001). The dried stem of *E. breviscapus* was extracted in a reflux apparatus (30% ethanol, 3 h at 85 °C), and the weight was 1 L: 100 g (30% EtOH: dried stem of *E. breviscapus*). A powder was produced by filtering, condensing, and spray drying the extract at low pressure. The yield of the extract was 23.26% (weight; 23.26 g). The extract was lyophilized under −80 °C.

### 2.2. High Performance Liquid Chromatography (HPLC) Analysis of EBE

For a component analysis of EBE, HPLC using an 1100 series (Agilent, Santa Clara, CA, USA) was utilized, and the conditions of the HPLC analysis are presented in Table 1.

### 2.3. Animal Treatment

The male Sprague-Dawley (SD) rat (190–210 g) and the male ICR mouse (28–32 g) were provided from DBL Co., Ltd. (DBL, Paju-si, Republic of Korea). Before the experiment, the animals were provided with at least seven days to become used to the common laboratory settings, which included 22 ± 2 °C, 55 ± 10% humidity, and a 12 h light/dark cycle. The animals were allowed to freely take water and food. The Gachon University Center of Animal Care and Use approved all of the experiments listed above (GU1-2022-IA0071-01).

### 2.4. Acetic Acid-Induced Peripheral Pain Mice Model

Four groups of eight Institute of Cancer Research (ICR) mice each were isolated, and they were given 200 and 600 mg/kg of EBE, 200 mg/kg of ibuprofen (Sigma, Marlborough, MA, USA), and distilled water as a control. As a positive control, ibuprofen was utilized. After 30 min of oral treatment, 10 mL/kg of 0.7% acetic acid was administered intraperitoneally, and 10 min later writhing reactions were recorded. A twist reaction was formed by contracting the abdominal wall and turning the pelvis in response to hind limb swelling.

### 2.5. MIA-Induced Osteoarthritis Rat Model

This experiment was designed to provide an animal model for MIA induction. Each of the five groups contained nine rats. The four groups were as follows: sham, indomethacin (INDO 3), control (CON), and EBE 300. After being sedated with a O_2_ mixture and 2% isofluorane, intraarticular injections 50 μL of 40 mg/mL MIA (Sigma Aldrich Inc., St. Louis, MO, USA) were used to generate OA in the CON, INDO 3, and EBE groups (Table 2). A 50 μL measure of saline was injected into the joint cavity of the knee to the Sham group. DW was given to the sham and CON groups every day for a duration of 24 days. During the 24 days, indomethacin (Sigma, USA) was given daily at a dose of 3 mg/kg P.O. after being dissolved in distilled water (DW). For 24 days, EBE 300 mg/kg was given orally once a day after being dissolved in DW. Each group received the samples P.O. to dissolve them in DW at 10 mL/kg. The mice were CO_2_-euthanized at the conclusion of the experiment.

### 2.6. Weight-Bearing Measurement

Using an incapacitance Meter Tester 600 (IITC Life Science Inc., Woodland Hills, CA, USA) after OA was induced in SD rats, the weight-bearing of the hind leg was recorded on days 0, 3, 7, 10, 14, 17, 21, and 24. The average strength supplied to each limb over a 10 s period was then calculated. The following calculation was utilized to calculate the percentage of weight distributed in the hind limb of the treated side (right).

$$Weight\text{-}bearing\;ratio\;\left(\%\right)\;=\;\frac{weight\;of\;right\;hind\;limb}{weight\;of\;right\;and\;left\;hind\;limbs}\;\times \;100$$

### 2.7. Cartilage Degradation Analysis

The rat was sacrificed, and the right-side knee joints were disarticulated and examined for macroscopic scoring. A digital Sony α6600 camera (Sony Corp., Tokyo, Japan) was used to take pictures of the MIA-induced model’s knee. A macroscopic scoring method was used to evaluate the degradation of the articular cartilage (Table 3).

### 2.8. Serum Measurement of OA Induced Model

To create a blood clot, whole blood was extracted from the abdomen vein and left for thirty minutes. The separated serum was kept at −70 °C after the samples were centrifuged for 10 min at 4000 rpm. A Premixed Multi-Analyte Kit (R&D Systems Inc., Minneapolis, MN, USA) was used to detect IL-1β and IL-6 levels in serum. Results were evaluated using a Luminex analyzer (Luminex Co., Madison, WI, USA). All multiplex test experiments were carried out in accordance with the manufacturer’s protocol.

### 2.9. Measurement of Cell Toxicity and NO Generation

RAW264.7 macrophages were purchased from the American Type Culture Collection (ATCC, Manassas, VA, USA). The cells were cultured in DMEM media supplemented with 10% FBS, 100 IU/mL penicillin- streptomycin (Gibco BRL, Carlsbad, CA, USA) at 37 °C and 5% CO_2_. The cells were seeded and then treated for 24 h with doses of 500 ng/mL LPS and EBE (10–300 µg/mL). The supernatant was combined with Greiss reagent, and the absorbance at 540 nm was recorded. Cytotoxicity was measured using the Ez-Cytox reagent (DoGenBio, Seoul, Republic of Korea). In summary, the Ez-Cytox reagent was used to analyze cell viability in accordance with the manufacturer’s protocol. This experiment was conducted three times.

(1) $$\%\;of\;control\;=\;\frac{each\;sample\;raw\;data}{average\;value\;of\;control}\;\times \;100$$

### 2.10. Analysis of Quantitative Real-Time Polymerase Chain Reaction (qRT-PCR)

Cartilage tissue-induced OA and LPS-induced RAW264.7 cells were used to extract total RNA by the AccuPrep^®^ RNAExtractionKit (Bioneer, Daejeon, Republic of Korea). CycleScript^TM^ RT Pre&Master Mix (Bioneer, Daejeon, Republic of Korea) was then used to reverse transcribe the extracted RNA into cDNA in accordance with the manufacturer’s procedure. Using the 2X-GreenStar^TM^ qPCR MasterMix (Bioneer, Republic of Korea), mRNA expression was measured. All tests were conducted three times. The threshold cycle (CT) approach was utilized to ascertain the relative gene expression, whereas the 2^−ΔΔCT^ formula was employed to compute the fold changes. The primer sequences, which were made up of exons, are shown in the tables below (Table 4 and Table 5).

### 2.11. Protein Expression

The protein expression of IL-1β, IL-6, Ptger2, NOS2, MMP1, MMP8, MMP13, and GAPDH was examined by Western blot analysis. OA-induced cartilage was homogenized with RIPA buffer (CST Inc., Kansas City, MO, USA) and cOmplete^TM^ Protease Inhibitor (Sigma, USA) to extract for protein. Using the Mini Trans-Blot^®^ Cell (BioRad, Hercules, CA, USA) at 15 V, the extracted proteins were transferred onto PVDF membranes for 45 min after which the proteins (10 μg) were put onto MiniPROTEAN^®^ TGX^TM^ Precast Gel (BioRad Laboratories, Inc., Hercules, CA, USA). Membranes were treated for 15 min at room temperature with EveryBlot Blocking Buffer (BioRad, USA) in order to prevent non-specific antibody binding. The primary antibodies (IL-1β, IL-6, Ptger2, NOS2, MMP1, MMP8, MMP13, and GAPDH) were incubated at 4 °C for 24 h. After one hour at room temperature, the membrane was probed with a secondary antibody and then reacted with Clarity^TM^ Western ECL Substrate (BioRad, USA) solution. Azure 280 was used to identify the Western blot image (Azure Biosystems, Dublin, CA, USA).

### 2.12. Statistics

The statistical analysis was performed using GraphPad Prism^®^ 9.0 (GraphPad Software, San Diego, CA, USA), which included a 1-way ANOVA with Dunnett’s post hoc test. The measurements were expressed as mean ± the standard deviation, with a significance level of *p* < 0.05.

## 3. Results

### 3.1. HPLC Analysis

**Figure 1:**
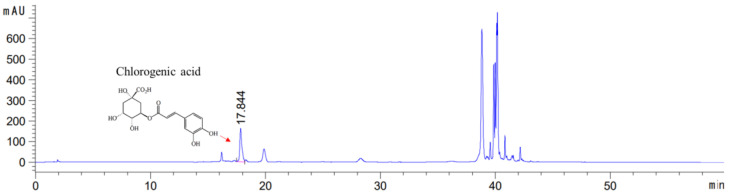
High-performance liquid chromatography chromatogram of the *Erigeron breviscapus* extract at 325 nm: Chlorogenic acid Retention time = 17.844 min. *x*-axis: retention time; *y*-axis: absorbance unit. A Triart C18 column (150 mm × 4.6 mm id, 5 μm) (YMC-PACK^®^, Japan) was utilized for chromatic separation. The pink line calculates the content by integrating the peaks of chlorogenic acid to get the area value.

The present study aimed to investigate the anti-inflammatory-based OA modulatory effects of EBE as a whole extract. Therefore, although other major active components of EBE have been previously reported to have potent bioactivity, only chlorogenic acid was identified in this HPLC for its anti-inflammatory effects. Chlorogenic acid was obtained and checked in EBE with the HPLC-UV method. The extract contained 7.50 mg/g of chlorogenic acid. Figure 1 indicates the molecular composition of the constituent compound as well as the HPLC chromatogram of the analysis.

### 3.2. Effect of Analgesic Onacetic Acid-Induced Peripheral Pain Mice Model

**Figure 2:**
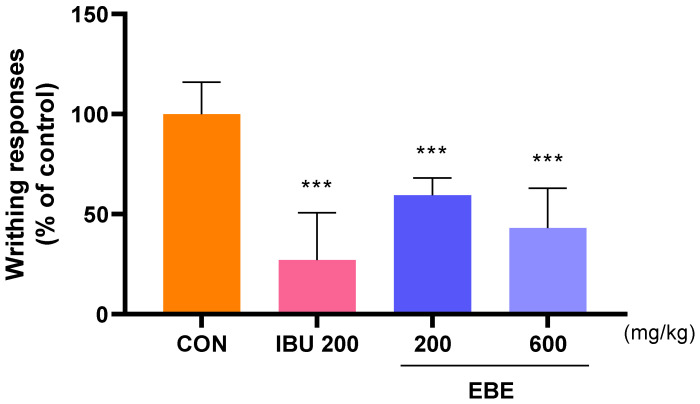
The number of writhing reactions in mice with acetic acid-induced peripheral pain mice model. Following 30 min of oral treatment, 0.7% acetic acid was intraperitoneally given into each mouse prior to a 10 min count. The number of mice was 8 per group; *** *p* < 0.001 vs. CON by 1 way ANOVA, Dunnett’s test. EBE: *Erigeron breviscapus* extract, IBU 200: ibuprofen 200 mg/kg.

The analgesic properties of EBE were researched according to the writhing response in acetic acid-induced peripheral pain mice. Mice with peripheral pain generated by acetic acid were seen to writhe in response to the analgesic action of EBE. The average number of writhing responses in the control group for 10 min was measured as 100. The number of writhing responses was lower after EBE treatment than it was for the control group. This result indicated the analgesic effects of EBE (Figure 2).

### 3.3. Effects on Weight-Bearing Distribution in MIA Rat

**Figure 3:**
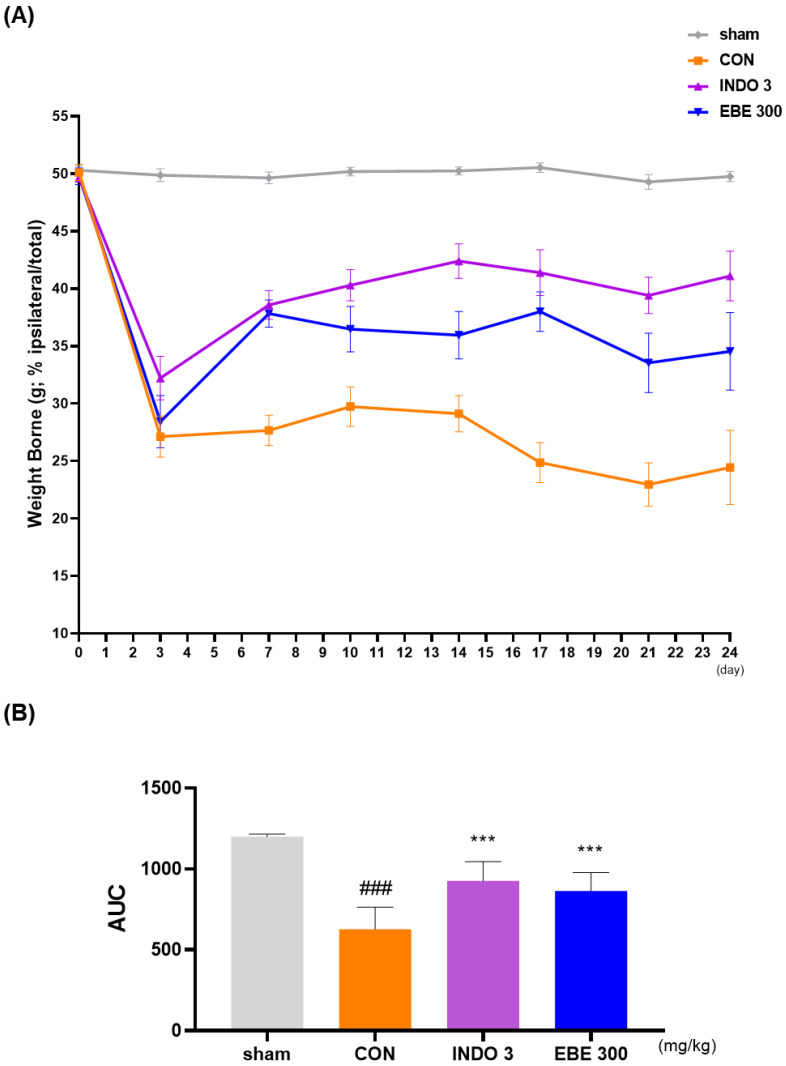
The effect of EBE on hind paw weight-bearing in a model of osteoarthritis caused by MIA. (**A**) The weight-bearing distribution of MIA rats was observed for a period of 0–24 days while they were treated with EBE 300 or INDO 3 (*n* = 9, per group). (**B**) An incapacitance meter tester was used to analyze the area under the curve (AUC). ### *p* < 0.001 vs. sham, *** *p* < 0.001 vs. control. CON: control, EBE: *Erigeron breviscapus*, INDO 3: 3 mg/kg of indomethacin, MIA: monosodium iodoacetate.

In MIA rats, the weight-bearing ratio of the hind leg was frequently utilized as an indicator of joint discomfort and pain in order to calculate the analgesic properties of natural products on OA. Twenty four days following OA induction, the weight-bearing ratio between the right and left legs was measured. In contrast to the sham group, the weight-bearing distribution in the control (MIA) group significantly decreased on day 7 and stayed lower, as shown in Figure 3A. In contrast, the administration of EBE led to a significant improvement in the weight-bearing capacity of MIA rats. In instance, the increase in weight-bearing caused by EBE was similar to that of INDO 3 (Figure 3B).

### 3.4. Effects on Knee Cartilage Damage in OA Rat Model

**Figure 4:**
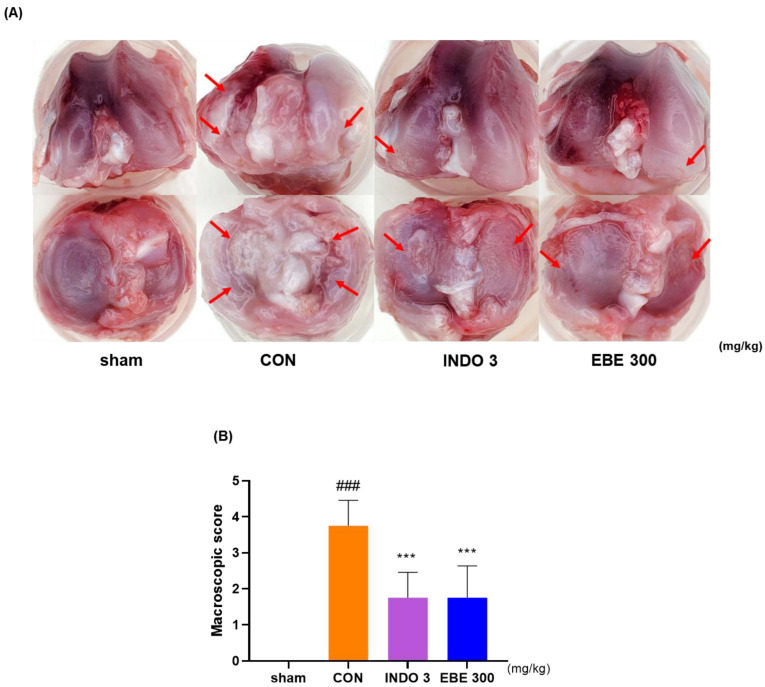
Photographs of the cartilages in the knee joints of rats with OA produced by MIA. (**A**) A representative photo of cartilage degradation after INDO 3 and EBE 300 was delivered to MIA rats. Arrows pointed to the cartilage degeneration site. (**B**) A macroscopic score. MIA rats were treated with INDO 3 and EBE 300 body weight (*n* = 9, per group). ### *p* < 0.001 vs. sham, *** *p* < 0.001 vs. control. CON: control, EBE: *Erigeron breviscapus* extract, INDO 3: 3 mg/kg of indomethacin, MIA: monosodium iodoacetate.

The representative photographs of knee cartilage in each experimental group indicate that EBE prevented cartilage erosion induced by MIA injection. As depicted in Figure 4, the knee cartilage of the sham group appeared lustrous and smooth, while that of the control group exhibited less polish and roughness, with damage evident in some areas. The cartilage erosion induced by CON was significantly mitigated in the EBE- and INDO 3-treated groups. The recovery of cartilage degradation by EBE was comparable to that of INDO 3.

### 3.5. Effect of Inflammatory Cytokines in MIA Rat Model

**Figure 5:**
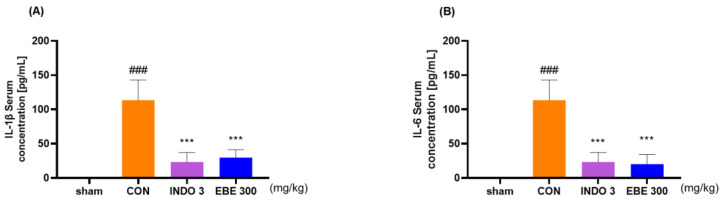
Serum inflammatory cytokine levels (**A**) IL-1β and (**B**) IL-6 in MIA rats. Rats were administered with EBE 300 for 24 days (*n* = 9, per group). ### *p* < 0.001 vs. sham *** *p* < 0.001 vs. CON by 1 way ANOVA, Dunnett’s test. CON: control, EBE: *Erigeron breviscapus* extract, INDO 3: 3 mg/kg of indomethacin, MIA: monosodium iodoacetate.

After separating the serum from blood drawn from each experimental group, the concentrations of IL-1β and IL-6 in the rats’ serum were measured. The group administered EBE showed a significant dose-dependent decrease in serum concentrations of IL-1β and IL-6 compared to the control group. Notably, EBE reduced the cytokine levels to a degree comparable to the positive control (Figure 5).

### 3.6. Effects on Cytokine Responses in Knee Cartilage Tissue

**Figure 6:**
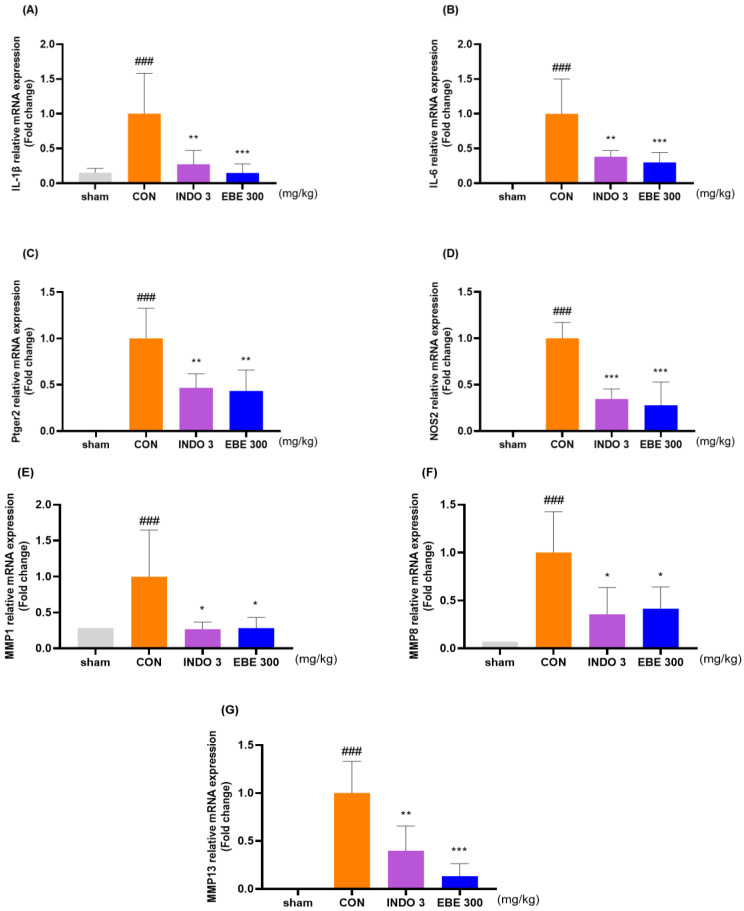

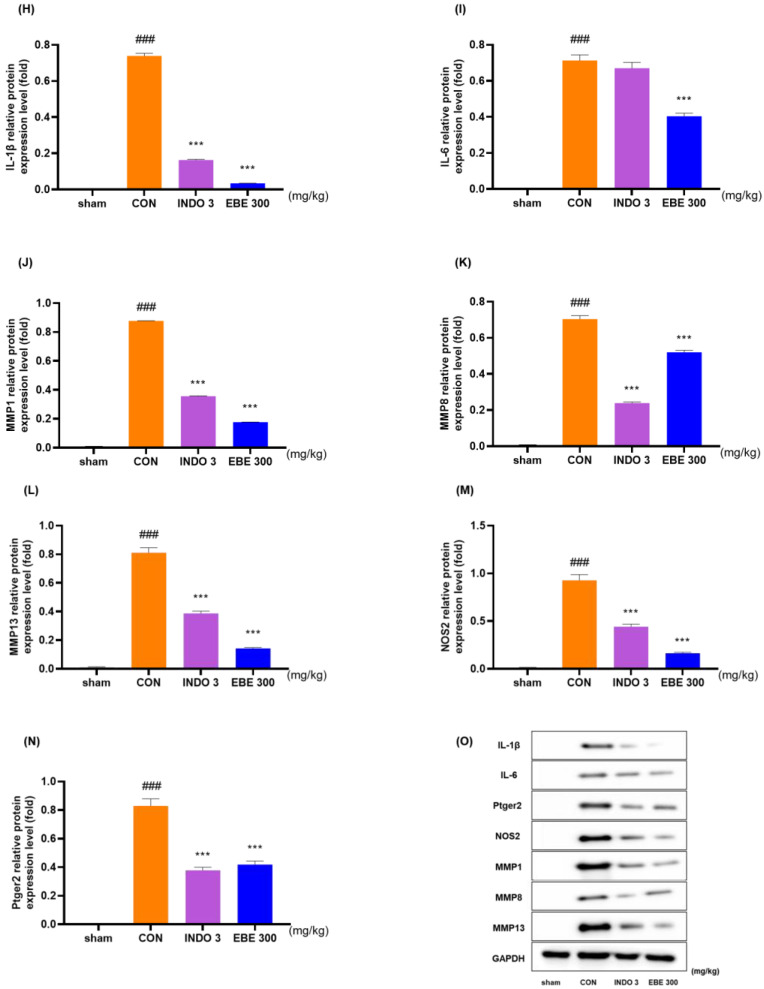
Cytokine changes in the cartilage tissue of the knee joint following EBE treatment. (**A**–**G**) Using quantitative real-time PCR, the mRNA expression of IL-1β, IL-6, ptger2, NOS2, MMP-1, MMP-8, and MMP-13 was ascertained. (**H**–**N**) Western blot analysis was used to evaluate the protein expression of IL-1β, IL-6, MMP-1, MMP-8, MMP-13, NOS2 and ptger2. (**O**) Western blot image. ### *p* < 0.001 vs. sham, * *p* < 0.05 vs. CON, ** *p* < 0.01 vs. CON, *** *p* < 0.001 vs. CON by 1 way ANOVA, Dunnett’s test. CON: control, EBE: *Erigeron breviscapus* extract, INDO 3: indomethacin 3 mg/kg, MIA: monosodium-iodoacetate.

The EBE group significantly lowered the levels of IL-1β, IL-6, ptger2, NOS2, MMP-1, MMP-8, and MMP-13 in knee cartilage, as compared to the control rats, according to the evaluation of the mRNA levels of these proteins in the rats (Figure 6A–G). The downregulating effects of EBE on IL-1β, IL-6, MMP-1, MMP-8, MMP-13, NOS2 and ptger2 were shown in MIA rats by western blot data (Figure 6H–N).

### 3.7. Anti-Inflammatory Effects in LPS-Induced RAW264.7 Cells

**Figure 7:**
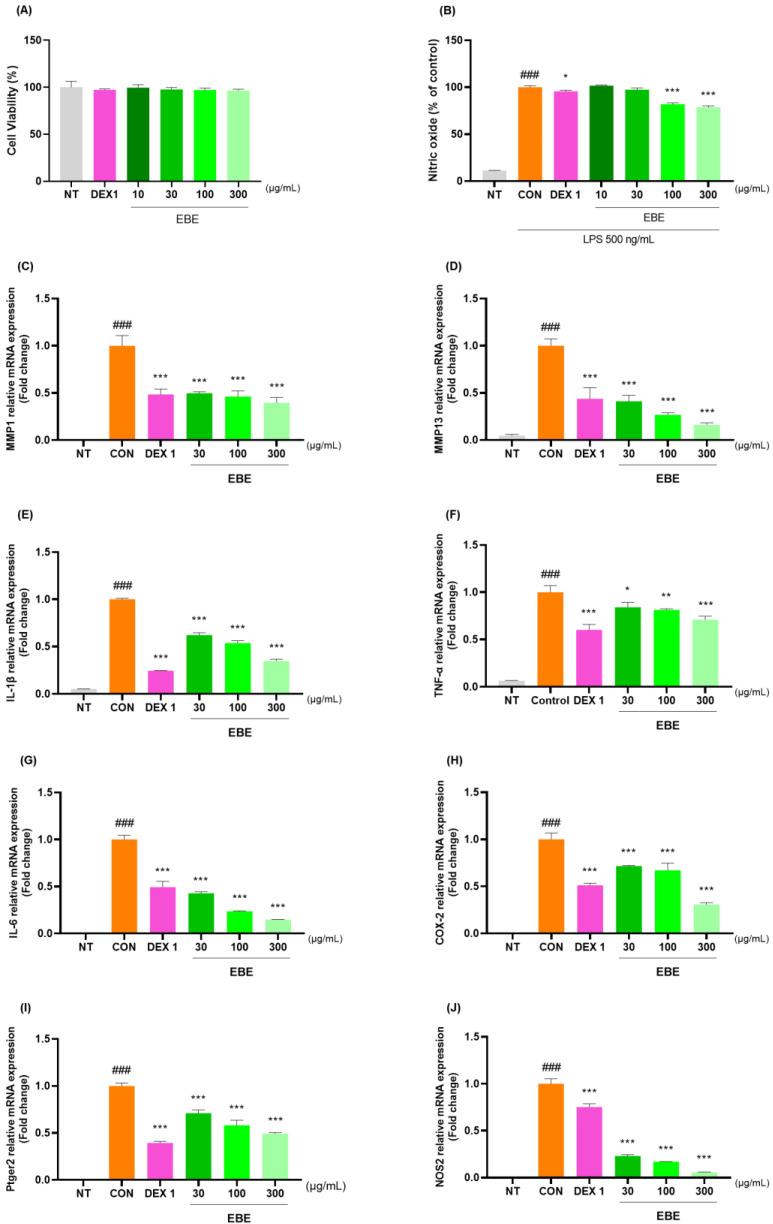

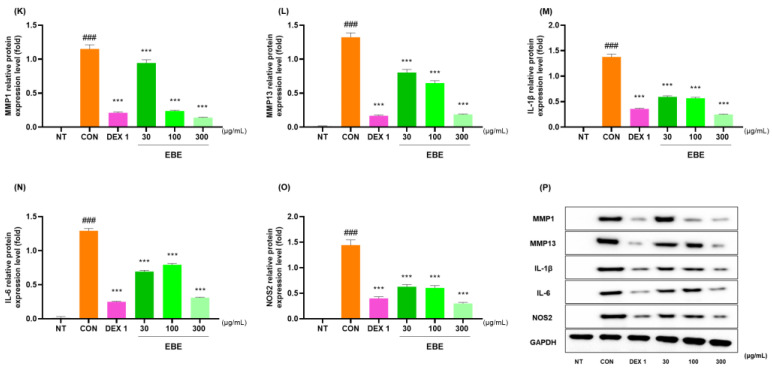
Effects of EBE on RAW264.7 (**A**) cell viability, (**B**) LPS-induced NO generation, (**C**–**J**) mRNA MMP-1, MMP-13, IL-1β, TNF-α, IL-6, COX-2, Ptger2, and NOS2, and (**K**–**P**) protein expression of MMP-1, MMP-13, IL-1β, TNF-α, IL-6, and NOS2. For a duration of 24 h, the cells were exposed to DEX 1, EBE 30, 100, and 300, and LPS. ### *p* < 0.001 vs. sham, * *p* < 0.05 vs. CON, ** *p* < 0.01 vs. CON, *** *p* < 0.001 vs. CON by a 1 way ANOVA, Dunnett’s test. CON: control, DEX 1: dexamethasone 1 µg/mL, EBE: *Erigeron breviscapus* extract, LPS: lipopolysaccharide, NO: nitric oxide, NT: non-treated, ANOVA: analysis of variance.

The anti-inflammatory properties of EBE were measured in LPS-treated RAW264.7 cells. At a concentration of 300 µg/mL, EBE showed no signs of possible cytotoxicity in RAW264.7 cells (Figure 7A). EBE reduced LPS-induced NO generation. When comparing the 300 EBE to the control, there was an NO decrease (Figure 7B). EBE reduced the levels of NO, demonstrating strong anti-inflammatory properties. Figure 7C–J illustrates how EBE and DEX 1 reduced the levels of MMP-1, MMP-13, IL-1β, TNF-α, IL-6, COX-2, Ptger2, and NOS2 mRNA expression. The anti-inflammatory properties of EBE in LPS-induced RAW264.7 cells were examined using Western blotting. As seen in Figure 7K–P, EBE administration of LPS-stimulated RAW264.7 cells suppressed the protein production of proinflammatory cytokines, including MMP-1, MMP-13, IL-1β, TNF-α, IL-6, and NOS2. EBE decreased the expression of MMP-1, MMP-13, IL-1β, TNF-α, IL-6, COX-2, Ptger2, and NOS2, as shown by the Western blot images. Notably, EBE showed comparable anti-inflammatory benefits to the positive control against all cytokines.

## 4. Discussion

The primary findings of this study suggest that *Erigeron breviscapus* (Vant.) Hand.-Mazz. extract (EBE) holds promise as a potential candidate for inhibiting inflammatory pain, alleviating functional decline, and preventing cartilage destruction in osteoarthritis (OA) owing to its anti-inflammatory properties. In an acetic acid-induced mouse model, EBE exhibited significant antinociceptive activity comparable to the active control ibuprofen, and this effect was dose-dependent. These analgesic and functional improvement effects were consistently validated through weight-bearing distribution measurements in an MIA-induced rat model. Furthermore, the inhibition of cartilage destruction in osteoarthritic rats treated with EBE was found to be comparable to that achieved with indomethacin. EBE also demonstrated significant downregulation of inflammatory cytokines in serum and knee cartilage tissue in the in vivo model. The anti-inflammatory activity of EBE in OA treatment was further supported by its ability to suppress the expression of various pro-inflammatory mediators, including IL-1β, IL-6, MMPs, and NOS2, in an in vitro model, with efficacy superior to or on par with the active control dexamethasone. To the best of our knowledge, this is the first study to explore the anti-OA activity of EBE based on its regulation of inflammatory pathology.

The symptomatic suppressive effect of EBE on the OA in vivo model is not surprising given the potential of this material. To date, EBE has a wide range of pharmacologic actions already identified in several previous studies, and there is an established evidence base of efficacy for many indications, mainly in cardiovascular and central nervous system diseases [20]. What is interesting about this research is that EBEs not only exert potent anti-inflammatory activity through a multitargeted mechanism of regulation of IL-1, IL-6, TNF-α, and others, but this pharmacologic activity has broad therapeutic implications for diseases of multiple systems in the body [21,22]. As previously mentioned, inflammatory stress contributes significantly to the development and progression of osteoarthritis pathology, including cartilage injury, joint inflammation, and bone remodeling, by disrupting chondrocyte survival, damaging the matrix, and promoting the production of pro-inflammatory mediators such as various matrix metalloproteinases and interleukins [23]. In this regard, numerous recent studies have demonstrated that the above inflammatory cytokines play a pivotal role in the process of chronic inflammation-induced OA inflammation, making them promising therapeutic targets [6]. Indeed, several clinical studies have shown that increased levels of inflammatory cytokines in older adults are strongly associated with increased morbidity and mortality in age-related diseases, including OA [24,25]. Despite this relatively well-established understanding of the inflammation-base pathology of OA and inflammatory cytokines as potential therapeutic targets, no single inhibitor against these targets has shown clinical efficacy as a DMOAD. This is likely because the inflammatory process in OA is a highly complex one that involves multiple inflammatory mediators and signaling pathways [26]. In this light, it is conceivable that the significant relief of OA symptoms such as pain, cartilage destruction, and functional disability exhibited by EBE is based on the multipharmacologic anti-inflammatory activity of the natural product against multiple targets. To further test this secondary hypothesis, the activity of EBE against several pro-inflammatory mediators was examined in this study utilizing both in vivo and in vitro models.

EBE significantly inhibited IL-1β and IL-6 in both serum and knee cartilage tissue of the MIA-induced OA rat model. As known in the literature, IL-1β is recognized as one of the most important inflammatory and catabolic cytokines in the pathophysiology of OA [27]. It accelerates cartilage degradation by increasing the expression and activity of matrix-degrading enzymes while interfering with the synthesis of major extracellular cartilage matrix components such as type II collagen and aggrecan. Therefore, inhibition of IL-1β-related pathways is expected to be an attractive target for treating the progression of cartilage destruction in OA. It is also noteworthy that IL-6 is the inflammatory cytokine most prominently associated with age-related diseases, including OA and its associated decline in physical function [6,28]. Recent studies have further solidified this link, as IL-6 signaling has been shown to contribute to both pain and cartilage destruction in post-traumatic OA [29]. Interestingly, this study reported that global IL-6 ablation led to an inhibition of calcitonin gene-related peptide (CGRP), a neurotransmitter in nociceptive nerves, and an inhibition of CRGP+ neuronal growth within the dorsal root ganglia in an animal model lumbar spine. In this vein, EBE’s inhibitory action on these two inflammatory cytokines, which not only promote the degenerative pathology of OA but are also directly involved in joint pain and physical deterioration, may partially explain the mechanisms underlying the relief of various OA symptoms shown by this material in in vivo models.

The inhibitory activity of EBE against several inflammatory mediators was also consistently confirmed in vitro. Of particular note, the effects on MMP13 and NOS2 were significantly greater than those of the active control, dexamethasone, with a clear dose-dependency. First discovered in the 1960s as collagen-degrading enzymes and named collagenase, MMPs have since been studied as major factors contributing to the degenerative pathology of OA and as prime therapeutic targets for drugs to control arthritis of various etiologies through their inhibition [30]. Of particular relevance to this study is that among several MMPs, MMP 13 has been implicated as an important factor in mechanical overload-induced chondrocyte senescence in osteoarthritis [30,31]. Both MMP1 and MMP13 are observed in the synovial fluid in pathologic situations such as cartilage breakdown, lymphocyte infiltration, and synovitis in OA, but MMP1 is also associated with rheumatoid arthritis, and MMP13 is the preferred therapeutic target for OA [32,33]. MMP13 is an enzyme that is increased by IL-1 and TNF in OA cartilage and synovial tissue and contributes significantly to collagen degradation. On the other hand, it is clear that MMP13 is the predominant type 2 collagenase in both animal models [34] and human OA [35]. The fact that MMP13 is also induced by non-inflammatory mechanisms, even when pro-inflammatory cytokine activity is not prominent, is an essential consideration in developing OA therapeutics [36]. As discussed, given that MMP13 is considered one of the most specific targets for the development of potential inhibitors for the treatment of OA based on a number of previous studies, the significant inhibitory efficacy of EBE against MMP13 in this study suggests that it may be an implicated finding in the further exploration of DMOADs candidates. On the other hand, the inhibitory effect on NOS2 is also of note because it is closely related to targets such as IL-1β and MMP13, which we discussed earlier. NOS is first aberrantly expressed in the surface area of cartilage, which is the initial site of damage in OA. NOS induces cartilage damage by increasing the activity of MMPs and downregulating aggrecan and collagen biosynthesis. In addition, IL-1β and TNF-α activate the NF-κB signaling pathway by reacting with NO to produce peroxynitrite, which promotes the expression of NOS [37]. As NOS functions as a crucial mediator in the OA pathogenesis, inhibition of NOS2 is also being investigated as an emerging pharmacological mechanism for potential DMOAD candidates. In a nutshell, the mechanisms of action demonstrated by EBE against key pro-inflammatory mediators in this study, both in vivo and in vitro, go beyond the mere confirmation of its excellent anti-inflammatory effects and provide some confirmation of the hypothesis that this material may be worthy of investigation as a DMOAD candidate that modulates the multiple pathologies of OA through its multitargeted activity.

As this study is part of the discovery and screening for natural product-based DMOAD candidates, it is not a hypothesis testing design and has several limitations. First, the HPLC analysis in this study only tested for chlorogenic acid, one of the 12 major bioactive compounds of EBE that have already been identified [38]. This is because chlorogenic acid has been reported to have a pronounced inhibitory effect on the pathology of OA, unlike the more extensively studied ingredient breviscapine [39]. On the other hand, scutellarin, another major individual component of EBE, was not included in the HPLC analysis in this study because it has been well established in the literature that it acts on multiple signaling pathways, including NF-κB, MAPK, Wnt, and PI3K/AKT/mTOR, and exhibits anti-OA activity consistent with that observed in this study [40,41,42,43,44]. In essence, our goal is not drug discovery for a single compound, and the effects observed in this study cannot be explained by the activity of single compound. Therefore, future studies will further investigate the multi-component and multi-target efficacy of EBE based on the prediction and validation of multiple important compounds using a merged design of network pharmacology techniques. Second, because this study was conducted for screening purposes, we did not examine the specific signaling pathways through which EBE’s anti-inflammatory activity is mediated in the treatment of OA. For that reason, we plan to further utilize bioinformatic techniques in future studies to identify the in-depth mechanism of action, including the key signaling pathways of EBE. Third, a prerequisite for EBE to be planned as a DMOAD candidate with potentially promising results is to confirm its effect on subchondral bone destruction and osteoporotic changes, which are critical progressive pathologies of OA that were not examined in this study. This will also be verified in follow-up experiments based on the results of this study. Finally, although some distinct dose-dependent effects were identified, the optimal dose of EBE could not be derived from this study. We recognize this as an ongoing issue that needs to be addressed in future experiments.

## 5. Conclusions

The present study demonstrates that EBE has a beneficial effect on clinical symptoms of OA, including pain, functional decline, and cartilage destruction, which may be related to its significant anti-inflammatory effects on pro-inflammatory mediators, including IL-1β, IL-6, MMP13, and NOS2, which directly contribute to the inflammatory pathophysiology of OA. Therefore, we suggest that EBE is a DMOAD candidate that may be worthy of further study to determine whether it efficiently modulates the complex inflammatory pathology of OA based on its multicomponent, multitargeted action. However, further studies in expanded experimental designs with different components and specific mechanisms will be required to confirm whether EBE is indeed a promising DMOAD candidate that can enter clinical trials.

## Figures and Tables

**Table 1:** Conditions for HPLC analysis.

	Conditions
Colum	Triart C_18_ column (4.6 × 150 mm, 5 µm, YMC-PACK^®^, Kyoto, Japan)
Mobile phase	(A) acetonitrile, (B) 0.3% formic acid
Flow rate	0–10 min, 1–1%
	10–13 min, 1–15%
	13–35 min, 15–15%
	35–45 min, 15–100%
	45–60 min, 100–1% solvent B
Injection volume	1.0 mL/min
Detection wavelength	325 nm
Temperature	30 °C

**Table 2:** MIA-induced OA model design.

Group	OA Model (50 μL, Intraarticular)	Sample (10 mL/kg, P.O.)
sham	saline	DW
CON	MIA 40 mg/mL	DW
INDO 3	MIA 40 mg/mL	indomethacin 3 mg/kg
EBE 300	MIA 40 mg/mL	EBE 300 mg/kg

**Table 3:** Macroscopic scoring for damaged cartilage.

Score	Cartilage Appearance
0	Typical appearance on the surface of cartilage
1	Mild fibrillation or a yellowish discoloration on the surface
2	Erosion affecting the cartilage’s middle or outer layers
3	Deep erosions that extend to the subchondral bone
4	Massive erosions and widespread exposure of subchondral bone

**Table 4:** Primer for cartilage tissue-induced OA.

IL-1β	F	AACTCAACTGTGAAATAGCAGC
	R	TCCACAGCCACAATGAGTG
IL-6	F	TCCGCAAGAGACTTCCAGC
	R	CCTCCGACTTGTGAAGTGG
Ptger2	F	TGTGTGTACTGTCCGTCTGC
	R	CAGGGATCCAGTCTCGGTGT
NOS2	F	AGTCAACTACAAGCCCCACG
	R	GCAGCTTGTCCAGGGATTCT
MMP-1	F	AACTTGGGTGAAGACGTCCA
	R	TCCTGTCACTTTCAGCCCAA
MMP-8	F	TCTGTTCTTCTTCCACACACAG
	R	GCAATCATAGTGGCATTCCT
MMP-13	F	ACCTTCTTCTTGTTGAGTTGGA
	R	CTGCATTTCTCGGAGTCTA
GAPDH	F	CTTGTGACAAAGTGGACATTGTT
	R	TGACCAGCTTCCCATTCTC

IL: interleukin, Ptger2: prostaglandin E receptor 2, NOS: nitric oxide synthase, MMP: matrix metalloproteinase, GAPDH: Glyceraldehyde 3-phosphate dehydrogenase.

**Table 5:** Primer for LPS-induced RAW264.7 cells.

MMP-1	F	ATGCCTAGCCTTCCTTTGCT
	R	TTCCAGGTATTTCCAGACTG
MMP-13	F	AACCAAGATGTGGAGTGCCT
	R	GACCAGACCTTGAAGGCTTT
IL-1β	F	CCAGCTTCAAATCTCGCAGC
	R	GTGCTCATGTCCTCATCCTGG
TNF-α	F	GAGAAGTTCCCAAATGGCCT
	R	AGCCACTCCAGCTGCTCCT
IL-6	F	CACTTCACAAGTCGGAGGCT
	R	CAAGTGCATCATCGTTGTTC
COX-2	F	ATCCATGTCAAAACCGTGGG
	R	TTGGGGTGGGCTTCAGCAG
Ptger2	F	CTGGTAACGGAATTGGTGC
	R	TGGCCAGACTAAAGAAGGTC
NOS2	F	ACCAAGATGGCCTGGAGGAA
	R	CCGACCTGATGTTGCCATTG
GAPDH	F	ATGGTGAAGGTCGGTGTG
	R	GCCGTGAGTGGAGTCATAC

MMP: matrix metalloproteinase, IL: interleukin, TNF: tumor necrosis factor, COX: cyclooxygenase, Ptger2: prostaglandin E receptor 2, NOS: nitric oxide synthase, GAPDH: Glyceraldehyde 3-phosphate dehydrogenase.

## Data Availability

All data is contained within the article.

## References

[1] Sharma L. (2021). Osteoarthritis of the Knee. N. Engl. J. Med..

[2] (2023). GBD 2021 Osteoarthritis Collaborators Global, Regional, and National Burden of Osteoarthritis, 1990–2020 and Projections to 2050: A Systematic Analysis for the Global Burden of Disease Study 2021. Lancet Rheumatol..

[3] De Roover A., Escribano-Núñez A., Monteagudo S., Lories R. (2023). Fundamentals of Osteoarthritis: Inflammatory Mediators in Osteoarthritis. Osteoarthr. Cartil..

[4] Gezer H.H., Ostor A. (2023). What Is New in Pharmacological Treatment for Osteoarthritis?. Best. Pract. Res. Clin. Rheumatol..

[5] Englund M. (2023). Osteoarthritis, Part of Life or a Curable Disease? A Bird’s-Eye View. J. Intern. Med..

[6] Bi J., Zhang C., Lu C., Mo C., Zeng J., Yao M., Jia B., Liu Z., Yuan P., Xu S. (2024). Age-Related Bone Diseases: Role of Inflammaging. J. Autoimmun..

[7] Sanchez-Lopez E., Coras R., Torres A., Lane N.E., Guma M. (2022). Synovial Inflammation in Osteoarthritis Progression. Nat. Rev. Rheumatol..

[8] Van Spil W.E., Kubassova O., Boesen M., Bay-Jensen A.-C., Mobasheri A. (2019). Osteoarthritis Phenotypes and Novel Therapeutic Targets. Biochem. Pharmacol..

[9] Gouda N.A., Alshammari S.O., Abourehab M.A.S., Alshammari Q.A., Elkamhawy A. (2023). Therapeutic Potential of Natural Products in Inflammation: Underlying Molecular Mechanisms, Clinical Outcomes, Technological Advances, and Future Perspectives. Inflammopharmacology.

[10] Su J., Yu M., Wang H., Wei Y. (2023). Natural Anti-Inflammatory Products for Osteoarthritis: From Molecular Mechanism to Drug Delivery Systems and Clinical Trials. Phytother. Res..

[11] Fang S., Zhang B., Xiang W., Zheng L., Wang X., Li S., Zhang T., Feng D., Gong Y., Wu J. (2024). Natural Products in Osteoarthritis Treatment: Bridging Basic Research to Clinical Applications. Chin. Med..

[12] Jo H.-G., Lee G.-Y., Baek C.Y., Song H.S., Lee D. (2020). Analgesic and Anti-Inflammatory Effects of Aucklandia Lappa Root Extracts on Acetic Acid-Induced Writhing in Mice and Monosodium Iodoacetate-Induced Osteoarthritis in Rats. Plants.

[13] Jo H.G., Baek C.Y., Kim D., Kim S., Han Y., Park C., Song H.S., Lee D. (2023). Network Analysis, In Vivo, and In Vitro Experiments Identified the Mechanisms by Which *Piper longum* L. [Piperaceae] Alleviates Cartilage Destruction, Joint Inflammation, and Arthritic Pain. Front. Pharmacol..

[14] Jo H.-G., Baek C.-Y., Song H.S., Lee D. (2024). Network Pharmacology and Experimental Verifications to Discover Scutellaria Baicalensis Georgi’s Effects on Joint Inflammation, Destruction, and Pain in Osteoarthritis. Int. J. Mol. Sci..

[15] Peng Y., Yang Z., Li J., Liu S. (2024). Research Progress on Nanotechnology of Traditional Chinese Medicine to Enhance the Therapeutic Effect of Osteoarthritis. Drug Deliv. Transl. Res..

[16] Li W., Yu L., Li W., Ge G., Ma Y., Xiao L., Qiao Y., Huang W., Huang W., Wei M. (2023). Prevention and Treatment of Inflammatory Arthritis with Traditional Chinese Medicine: Underlying Mechanisms Based on Cell and Molecular Targets. Ageing Res. Rev..

[17] Jo H.-G., Baek C.Y., Kim D., Lee D., Song H.S. (2023). Stem of *Sorbus commixta* Hedl. Extract Inhibits Cartilage Degradation and Arthritic Pain in Experimental Model via Anti-Inflammatory Activity. Nutrients.

[18] Zhang R., Han L., Lin W., Ba X., Yan J., Li T., Yang Y., Huang Y., Huang Y., Qin K. (2024). Mechanisms of NLRP3 Inflammasome in Rheumatoid Arthritis and Osteoarthritis and the Effects of Traditional Chinese Medicine. J. Ethnopharmacol..

[19] Fan H., Lin P., Kang Q., Zhao Z.-L., Wang J., Cheng J.-Y. (2021). Metabolism and Pharmacological Mechanisms of Active Ingredients in *Erigeron breviscapus*. Curr. Drug Metab..

[20] Wu R., Liang Y., Xu M., Fu K., Zhang Y., Wu L., Wang Z. (2021). Advances in Chemical Constituents, Clinical Applications, Pharmacology, Pharmacokinetics and Toxicology of *Erigeron breviscapus*. Front. Pharmacol..

[21] Gao J., Chen G., He H., Liu C., Xiong X., Li J., Wang J. (2017). Therapeutic Effects of Breviscapine in Cardiovascular Diseases: A Review. Front. Pharmacol..

[22] Dong X., Qu S. (2022). *Erigeron breviscapus* (Vant.) Hand-Mazz.: A Promising Natural Neuroprotective Agent for Alzheimer’s Disease. Front. Pharmacol..

[23] Muthu S., Korpershoek J.V., Novais E.J., Tawy G.F., Hollander A.P., Martin I. (2023). Failure of Cartilage Regeneration: Emerging Hypotheses and Related Therapeutic Strategies. Nat. Rev. Rheumatol..

[24] Rea I.M., Gibson D.S., McGilligan V., McNerlan S.E., Alexander H.D., Ross O.A. (2018). Age and Age-Related Diseases: Role of Inflammation Triggers and Cytokines. Front. Immunol..

[25] Guo J., Huang X., Dou L., Yan M., Shen T., Tang W., Li J. (2022). Aging and Aging-Related Diseases: From Molecular Mechanisms to Interventions and Treatments. Signal Transduct. Target. Ther..

[26] Liu-Bryan R., Terkeltaub R. (2015). Emerging Regulators of the Inflammatory Process in Osteoarthritis. Nat. Rev. Rheumatol..

[27] Jenei-Lanzl Z., Meurer A., Zaucke F. (2019). Interleukin-1β Signaling in Osteoarthritis—Chondrocytes in Focus. Cell Signal.

[28] Singh T., Newman A.B. (2011). Inflammatory Markers in Population Studies of Aging. Ageing Res. Rev..

[29] Liao Y., Ren Y., Luo X., Mirando A.J., Long J.T., Leinroth A., Ji R.-R., Hilton M.J. (2022). Interleukin-6 Signaling Mediates Cartilage Degradation and Pain in Posttraumatic Osteoarthritis in a Sex-Specific Manner. Sci. Signal.

[30] Grillet B., Pereira R.V.S., Van Damme J., Abu El-Asrar A., Proost P., Opdenakker G. (2023). Matrix Metalloproteinases in Arthritis: Towards Precision Medicine. Nat. Rev. Rheumatol..

[31] Billinghurst R.C., Dahlberg L., Ionescu M., Reiner A., Bourne R., Rorabeck C., Mitchell P., Hambor J., Diekmann O., Tschesche H. (1997). Enhanced Cleavage of Type II Collagen by Collagenases in Osteoarthritic Articular Cartilage. J. Clin. Investig..

[32] Ulivi V., Giannoni P., Gentili C., Cancedda R., Descalzi F. (2008). P38/NF-κB-Dependent Expression of COX-2 during Differentiation and Inflammatory Response of Chondrocytes. J. Cell Biochem..

[33] Dahlberg L., Billinghurst R.C., Manner P., Nelson F., Webb G., Ionescu M., Reiner A., Tanzer M., Zukor D., Chen J. (2000). Selective Enhancement of Collagenase-Mediated Cleavage of Resident Type II Collagen in Cultured Osteoarthritic Cartilage and Arrest with a Synthetic Inhibitor That Spares Collagenase 1 (Matrix Metalloproteinase 1). Arthritis Rheum..

[34] Little C.B., Barai A., Burkhardt D., Smith S.M., Fosang A.J., Werb Z., Shah M., Thompson E.W. (2009). Matrix Metalloproteinase 13-Deficient Mice Are Resistant to Osteoarthritic Cartilage Erosion but Not Chondrocyte Hypertrophy or Osteophyte Development. Arthritis Rheum..

[35] Hu Q., Ecker M. (2021). Overview of MMP-13 as a Promising Target for the Treatment of Osteoarthritis. Int. J. Mol. Sci..

[36] Baral P., Udit S., Chiu I.M. (2019). Pain and Immunity: Implications for Host Defence. Nat. Rev. Immunol..

[37] Ahmad N., Ansari M.Y., Haqqi T.M. (2020). Role of iNOS in Osteoarthritis: Pathological and Therapeutic Aspects. J. Cell Physiol..

[38] Tian Y., Li Q., Zhou X., Pang Q., Xu Y. (2017). A UHPLC-MS/MS Method for Simultaneous Determination of Twelve Constituents from *Erigeron breviscapus* Extract in Rat Plasma: Application to a Pharmacokinetic Study. J. Chromatogr. B Analyt Technol. Biomed. Life Sci..

[39] Chen W.-P., Tang J.-L., Bao J.-P., Hu P.-F., Shi Z.-L., Wu L.-D. (2011). Anti-Arthritic Effects of Chlorogenic Acid in Interleukin-1β-Induced Rabbit Chondrocytes and a Rabbit Osteoarthritis Model. Int. Immunopharmacol..

[40] Wang W., Li J., Li F., Peng J., Xu M., Shangguan Y., Li Y., Zhao Y., Qiu C., Qu R. (2019). Scutellarin Suppresses Cartilage Destruction in Osteoarthritis Mouse Model by Inhibiting the NF-κB and PI3K/AKT Signaling Pathways. Int. Immunopharmacol..

[41] Luo Z., Hu Z., Bian Y., Su W., Li X., Li S., Wu J., Shi L., Song Y., Zheng G. (2020). Scutellarin Attenuates the IL-1β-Induced Inflammation in Mouse Chondrocytes and Prevents Osteoarthritic Progression. Front. Pharmacol..

[42] Liu F., Li L., Lu W., Ding Z., Huang W., Li Y.T., Cheng C., Shan W.S., Xu J., He W. (2020). Scutellarin Ameliorates Cartilage Degeneration in Osteoarthritis by Inhibiting the Wnt/β-Catenin and MAPK Signaling Pathways. Int. Immunopharmacol..

[43] Yang H., Wang Z., Wang L., Li Y., Guo J., Yang X., Zhao J., Rong K., Zhang P., Ye B. (2022). Scutellarin Ameliorates Osteoarthritis by Protecting Chondrocytes and Subchondral Bone Microstructure by Inactivating NF-κB/MAPK Signal Transduction. Biomed. Pharmacother..

[44] Ju S.-H., Tan L.-R., Liu P.-W., Tan Y.-L., Zhang Y.-T., Li X.-H., Wang M.-J., He B.-X. (2021). Scutellarin Regulates Osteoarthritis in Vitro by Inhibiting the PI3K/AKT/mTOR Signaling Pathway. Mol. Med. Rep..

